# Transportable data from non-target arthropod field studies for the environmental risk assessment of genetically modified maize expressing an insecticidal double-stranded RNA

**DOI:** 10.1007/s11248-015-9907-3

**Published:** 2015-10-03

**Authors:** Aqeel Ahmad, Ignacio Negri, Wladecir Oliveira, Christopher Brown, Peter Asiimwe, Bernard Sammons, Michael Horak, Changjian Jiang, David Carson

**Affiliations:** Monsanto Company, 800 N. Lindbergh Boulevard, St. Louis, MO 63141 USA; Monsanto Company, Fontezuela Research Station Route 8, km214, CP2700 Pergamino, Buenos Aires Argentina; Monsanto Company, Dionisio Bortolotti Avenue, km 0.5, Caixa Postal 9, Santa Cruz das Palmeiras, São Paulo Brazil

**Keywords:** Genetically modified crop, Insecticidal double-stranded RNA, *Bacillus thuringiensis*, Non-target arthropods, Environmental risk assessment, Transportability

## Abstract

As part of an environmental risk assessment, the potential impact of genetically modified (GM) maize MON 87411 on non-target arthropods (NTAs) was evaluated in the field. MON 87411 confers resistance to corn rootworm (CRW; *Diabrotica* spp.) by expressing an insecticidal double-stranded RNA (dsRNA) transcript and the Cry3Bb1 protein and tolerance to the herbicide glyphosate by producing the CP4 EPSPS protein. Field trials were conducted at 14 sites providing high geographic and environmental diversity within maize production areas from three geographic regions including the U.S., Argentina, and Brazil. MON 87411, the conventional control, and four commercial conventional reference hybrids were evaluated for NTA abundance and damage. Twenty arthropod taxa met minimum abundance criteria for valid statistical analysis. Nine of these taxa occurred in at least two of the three regions and in at least four sites across regions. These nine taxa included: aphid, predatory earwig, lacewing, ladybird beetle, leafhopper, minute pirate bug, parasitic wasp, sap beetle, and spider. In addition to wide regional distribution, these taxa encompass the ecological functions of herbivores, predators and parasitoids in maize agro-ecosystems. Thus, the nine arthropods may serve as representative taxa of maize agro-ecosystems, and thereby support that analysis of relevant data generated in one region can be transportable for the risk assessment of the same or similar GM crop products in another region. Across the 20 taxa analyzed, no statistically significant differences in abundance were detected between MON 87411 and the conventional control for 123 of the 128 individual-site comparisons (96.1 %). For the nine widely distributed taxa, no statistically significant differences in abundance were detected between MON 87411 and the conventional control. Furthermore, no statistically significant differences were detected between MON 87411 and the conventional control for 53 out of 56 individual-site comparisons (94.6 %) of NTA pest damage to the crop. In each case where a significant difference was observed in arthropod abundance or damage, the mean value for MON 87411 was within the reference range and/or the difference was not consistently observed across collection methods and/or sites. Thus, the differences were not representative of an adverse effect unfamiliar to maize and/or were not indicative of a consistent plant response associated with the GM traits. Results from this study support a conclusion of no adverse environmental impact of MON 87411 on NTAs compared to conventional maize and demonstrate the utility of relevant transportable data across regions for the ERA of GM crops.

**Electronic supplementary material:**

The online version of this article (doi:10.1007/s11248-015-9907-3) contains supplementary material, which is available to authorized users.

## Introduction

Prior to commercialization of a genetically modified (GM) crop, a science-based environmental risk assessment (ERA) is conducted to assess for potential harmful effects on human and animal health, and the environment. This process has been described in detail by a number of regulatory agencies worldwide [e.g., USDA-APHIS (CFR 2008), the U.S. Environmental Protection Agency (US EPA 1998), the Canadian Food Inspection Agency (CFIA 2012), and the European Union (EFSA 2004)]. For insect-protected GM crops, a step-wise, tiered testing approach using surrogate species within the framework of problem formulation is recognized as the most appropriate and rigorous approach to assess for potential effects on non-target organisms in many regulatory frameworks (Rose 2006; US EPA 2007; Romeis et al. 2008; Wolt et al. 2010). In this tiered approach, risk (a function of hazard and exposure) is evaluated within different levels or “tiers” that progress from worst-case exposure scenarios to real-world field scenarios if the earlier tiered tests fail to indicate adequate certainty of acceptable risk (Romeis et al. 2008; Duan et al. 2010).

In the ERA of GM crops, plant characterization studies are also conducted under diverse geographic and environmental conditions to assess potentially adverse effects of the GM crops on its receiving environment, relative to an appropriate conventional control that is genetically similar but lacks the introduced trait (Raybould 2007; Horak et al. 2007; Nickson 2008; Raybould 2010; Wolt et al. 2010; Horak et al. 2015a, b). These studies are used by risk assessors and regulators to determine whether cultivation and/or import of a GM crop is acceptable in a particular region.

Non-target arthropod (NTA) field evaluations are conducted when needed as an important part of plant characterization and are utilized in an overall ERA of the GM crop. The purpose of these evaluations is to confirm the results of the early tier testing and address any uncertainties in the risk assessment by collecting meaningful data on NTAs that are closely associated with the plant (Romeis et al. 2006, 2008). NTAs are selected based on criteria that they are sufficiently abundant in the crop of interest, exhibit low mobility and possess a clear path of exposure (e.g., non-target herbivores) (Prasifka et al. 2008; Romeis et al. 2009; Rauschen et al. 2010a, b; Romeis et al. 2013). Results from these evaluations, which may be considered higher tier, “real-world” assessments, aid in the ERA to reduce uncertainty of unintended effects through collection of *in planta* data. While NTA field data for plant characterization may be confirmatory of the tiered approach, a key distinction between the environmental interactions assessment and a higher-tier NTA field study is that the latter is conducted only if results from lower-tier laboratory NTA testing fail to indicate acceptable environmental risk for the GM crop product.

It is important that risk assessors and regulators have access to and utilize environmental assessment data on the crop and trait that are generated in other relevant geographic regions (Roberts et al. 2014; Garcia-Alonso et al. 2014; Horak et al. 2015a, b). The results from well-designed studies conducted in the field, greenhouse, or laboratory and used for ecological risk assessments are relevant and transportable to other geographies for the ERA of the same GM crop, or related traits or GM crop/trait combinations where the ecological assessment endpoints are similar. Leveraging existing, relevant data for the ERA of GM crops across regions will conserve resources, eliminate redundancy, and support conclusions with high certainty for assessing potential environmental risk from the commercial release of a GM crop.

Monsanto Company has developed GM maize, MON 87411 that confers resistance to corn rootworm (CRW; *Diabrotica* spp.) and tolerance to the herbicide glyphosate. MON 87411 contains a suppression cassette that expresses an inverted repeat sequence designed to match a partial sequence of the *Snf7* gene from western corn rootworm (WCR; *Diabrotica virgifera virgifera*). The expression of the suppression cassette results in the formation of a double stranded RNA (dsRNA) transcript containing a 240 bp fragment of the WCR *Snf7* gene (DvSnf7) (Bolognesi et al. 2012). Upon consumption, the plant-produced dsRNA in MON 87411 is recognized by the CRW’s RNA interference (RNAi) machinery resulting in down-regulation of the targeted DvSnf7 gene leading to CRW mortality (Bolognesi et al. 2012). MON 87411 also contains a Cry3Bb1 gene that produces a modified *Bacillus thuringiensis* (*Bt*) (subsp. *kumamotoensis*) Cry3Bb1 protein to protect against CRW larval feeding. In lab studies, the snf7 ortholog has been shown to have a very specific and narrow spectrum of activity limited to the Galerucinae subfamily of Chrysomelidae (Bachman et al. 2013). In addition, MON 87411 contains the *cp4 epsps* gene from *Agrobacterium* sp. strain CP4 that encodes for the 5-enolpyruvylshikimate-3-phosphate synthase (EPSPS) protein, which confers tolerance to glyphosate, the active ingredient in Roundup^®^ agricultural herbicides. MON 87411 builds upon the current *Bt* protein-based mode-of-action (MOA) for CRW control by the addition of a new RNAi-based MOA that offers enhanced control of target insect pests and prolonged durability of existing *Bt* technologies designed to control CRW.

Several studies have demonstrated the absence of adverse effects of crops expressing *Bt* proteins on non-target arthropods in the lab or field (Li and Romeis 2009, 2010, 2011; Ahmad et al. 2005, 2006; Bhatti et al. 2005a, b; Naranjo et al. 2005; Naranjo 2009; Marvier et al. 2007; Duan et al. 2008a, b; Rauschen et al. 2010a, b; Rosca and Cagan 2012a, b; Comas et al. 2014), however no published study has evaluated the effect of an RNA-based trait stacked with *Bt* proteins on abundance of NTAs in the field. This study evaluated the effect of MON 87411 on the abundance of NTAs relative to its conventional control in maize fields in three separate geographic regions, the U.S., Argentina, and Brazil. In addition, plant damage from major non-target pests was evaluated to determine whether MON 87411 had any increased or decreased susceptibility to these pests, providing more information on potential harmful effects for the ERA. Since the studies are conducted in diverse geographic regions representing a broad range of environmental conditions and agricultural ecosystems, and given the similarity of the endpoints being assessed, these results could be ‘‘transportable’’ to other countries. This paper also provides data supporting the concept of data transportability, where results on NTA data with proper justification can be leveraged across regions to support ERA.

## Materials and methods

### Study sites and materials

Data were collected from field trials conducted at four sites in the U.S. during the 2012 season, four sites in Argentina during the 2012–2013 season, and six sites in Brazil during the 2013–2014 season. These field sites provided a range of environmental and agronomic conditions representative of commercial maize production in all three regions. At each site, MON 87411, the conventional control, and four commercial conventional reference hybrids were planted in a randomized complete block design with four replications. The control material has a genetic background similar to MON 87411 with the exception of the insect-protected and glyphosate tolerant traits; it does not contain the inserted genes present in MON 87411. The reference hybrids were commercially available and varied by site and study, thereby providing a range of values common to commercial maize for the assessed characteristics. Details on all study sites are given in Table 1. At each site, the entire study area was treated with the same agronomic inputs (e.g. fertilizer, irrigation, pesticides) to ensure uniform agronomic conditions.

**Table 1:** Description of Field Sites Used to Evaluate MON 87411

Site^1^	Planting date^2^	Harvest date^2^	Planting rate (seeds/m)	Plot Size (m × m)	Soil type	% OM^3^	Previous crop
USA							
IABG	05/09/12	10/05/12	7.2	6.1 × 12.2	Loam	4.0	Soybean
NCBD	05/11/12	09/20/12	6.6	6.1 × 15.5	Sandy Loam	2.6	Cotton
NEYO	05/08/12	10/09/12	7.2	6.1 × 12.2	Silt Loam	3.0	Soybean
PAHM	05/19/12	10/19/12	8.2	6.1 × 12.2	Loam	1.6	Vegetables^4^
Argentina							
BAFE	12/11/12	05/10/13	6.0	9.8 × 10	Loam	3	Corn
BAGH	12/12/12	05/21/13	7.0	9.8 × 10	Silt Loam	2.6	Soybeans
ERMY	01/07/13	06/18/13	7.0	7.28 × 10	Silt Loam	3.5	Soybeans
TMBU	01/20/13	06/03/13	6.0	7.28 × 10	Loam	3.8	Wheat
Brazil							
BALM	11/24/13	04/07/14	7.0	6.4 × 5.0	Sand	1.7	Fallow
MGCH	11/14/13	04/04/14	7.0	6.4 × 5.0	Loam	2.5	Soybeans
MTSO	11/22/13	03/18/14	7.0	6.4 × 5.0	Loam	3.0	Fallow
PRRO	11/14/13	04/23/14	7.0	6.4 × 5.0	Loam	2.2	Oat
RSNM	11/24/13	04/17/14	7.0	6.4 × 5.0	Loam	2.7	Oat
SPSD	11/13/13	04/09/14	7.0	6.4 × 5.0	Loan	2.8	Millet

^1^ Site code: IABG = Greene County, IA; NCBD = Perquimans County, NC; NEYO = York County, NE; PAHM = Berks County, PA. BAFE = Ferré, Buenos Aires; BAGH = Gahan, Buenos Aires; ERMY = Montoya, Entre Ríos; TMBU = Burruyacú, Tucumán; BALM = Luis Eduardo Magalhães, BA; MGCH = Cachoeira Dourada, MG; MTSO = Sorriso, MT; PRRO = Rolândia, PR; RSNM = Não-Me-Toque, RS; SPSD = Santa Cruz das Palmeiras, SP

^2^ Planting and Harvest Date = mm/dd/yy

^3^ % OM = Percent Organic Matter

^4^ Vegetables = peppers, tomatoes, potatoes, cabbage, maize

### NTA abundance and damage assessments

#### Sticky traps

Arthropods were collected using yellow sticky traps (Pherocon AM, no-bait sticky traps; Great Lakes Integrated Pest Management, Vestaburg, MI) at five times during the growing season: late vegetative-VT, R1, R2, R3, and R4 growth stage (U.S. and Argentina) and V13–V15, VT–R1, R1–R2, R2–R3 and R3–R4 growth stage (Brazil). In each plot, sticky traps (two per plot in U.S. and Argentina; four per plot in Brazil) were deployed for approximately 7 days at the approximate midpoint between the ground level and the top of the plant canopy. Arthropods collected from sticky traps were identified and enumerated by skilled personnel/entomologists.

#### Visual counts

Visual counts were conducted at 5–6 times during the growing season: late vegetative, VT-R1, R1, R2, R3, and R4–R5 (U.S. and Argentina) and V13–V15, VT–R1, R1–R2, R2–R3 and R3–R4 (Brazil) from 5 to 10 non-systematically selected plants per plot. Visual counts for arthropod abundance were made by examining the stalk, leaf blade, leaf collar, ear tip, silk, and tassel of each plant.

#### Arthropod damage

In the U.S., damage from two non-target arthropod pests: *Helicoverpa zea* and *Ostrinia nubilalis*, was evaluated. Ear damage from *H. zea* was assessed at R5 by examining ten plants from two rows. Where damage was present, assessment was made using a plastic film grid (0.5 cm^2^ per grid) placed over the damaged area and counting the number of grid cells containing 50 % or greater damage. *O. nubilalis* damage was evaluated at R6 growth stage by splitting the stalk of 10 plants from two rows and recording the number and total length (cm) of all feeding galleries.

In Argentina and Brazil, damage from three non-target arthropod pests; *H. zea*, *Diatraea saccharalis*, and *Spodoptera frugiperda*, was evaluated. Ear damage from *H. zea* (Argentina) and Lepidopteran Insects, *H. zea* and *S. frugiperda* (Brazil) was assessed at R5–R6 using the methods described above for the U.S. study. *D. saccharalis* damage was evaluated at R6 growth stage by splitting the stalk of 10 plants from two rows and recording the number and total length (cm) of all feeding galleries. Leaf damage from *S. frugiperda* was evaluated up to 5 times, when larvae were actively causing damage, using a 0–9 Davis scale (Davis et al. 1992).

### Data analysis

#### Arthropod abundance data

The primary focus of the study was on the effects of MON 87411 and the conventional control on the mean count of each arthropod during the entire season in each region. In order to conduct a valid analysis of the material effect on arthropod counts, a two-part inclusion criteria was used. To meet the inclusion criteria for analysis, a mean count across all collection times per plot ≥ 1 was required for each site to be included in the analysis. Secondly, an average of at least one capture per replicate was required for each collection time to meet the standard for inclusion criteria. Data combinations with counts below these criteria were excluded from significance testing but summarized in Supplementary material. Two separate analyses were performed for the arthropod abundance data:

- An across-collection analysis was performed separately for each combination of collection method, arthropod taxa, region, and site using the following model:

1 $$y_{ijk} = \mu + B_{i} + M_{j} + \left( {BM} \right)_{ij} + C_{k} + \left( {BC} \right)_{ik} + \left( {MC} \right)_{jk} + e_{ijk}$$

where *y*_*ijk*_ = square-root of the observed count; *μ* = overall mean; *B*_*i*_ = random replicate effect; *M*_*j*_  = fixed material effect; (*BM*)_*ij*_ = random interaction of replicate and material; *C*_*k*_  = random collection effect; (*BC*)_*ik*_ = random interaction of replicate and collection; (*MC*)_jk_  = random interaction effect of material and collection; and *e*_*ijk*_ = residual effect. PROC MIXED using SAS^®^ (SAS 2008, 2012) was used to fit model (1) to the data. Heterogeneous variance was assumed to accommodate the observed heterogeneity among collections. A square-root variance stabilizing transformation was used to account for the count nature of the data. Pairwise comparisons between MON 87411 and conventional control materials were defined within the ANOVA and tested using *t* tests.

(b) An across-region-site-collection analysis was performed for insects captured in at least two regions using the following model:

2 $$y_{ijklm} = \mu + R_{i} + S_{j\left( i \right)} + B_{{k\left( {ij} \right)}} + M_{l} + \left( {RM} \right)_{il} + \left( {BM} \right)_{{kl\left( {ij} \right)}} + \left( {SM} \right)_{jl\left( i \right)} + C_{{m\left( {ij} \right)}} + \left( {BC} \right)_{{km\left( {ij} \right)}} + \left( {MC} \right)_{{lm\left( {ij} \right)}} + e_{ijklm}$$

where *y*_*ijklm*_ = square-root of the observed count; *μ* = overall mean; *R*_*i*_ = fixed region effect; *S*_*j*(*i*)_ = random site effect within region; *B*_*k*(*ij*)_ = random replicate (block) effect within each site; *M*_*l*_ = fixed material effect; (*RM*)_*il*_ = fixed interaction of region and material; $\left( {BM} \right)_{{kl\left( {ij} \right)}}$  = random interaction of replicate and material; *C*_*m*(*ij*)_ = random collection effect within each site; $\left( {SM} \right)_{jl\left( i \right)}$  = random interaction of material and site within region; (*BC*)_*km*(*ij*)_ = random interaction of replicate and collection;$\left( {MC} \right)_{{lm\left( {ij} \right)}}$  = random interaction of material and collection time; and *e*_*ijklm*_ = residual effect. This model is essentially the same as model (1) above for each site except for the addition of the fixed effects of region and its interaction with the material and random site effects within each region. PROC MIXED using SAS^®^ (SAS 2008, 2012) was used to fit model (2) to nine insects with data from at least two regions reaching the site-inclusion criteria. Pairwise comparisons between MON 87411 and conventional control materials were defined within the ANOVA and tested using *t* tests.

#### Arthropod damage data

A combined-site ANOVA was conducted according to the following model for a randomized complete block design:

3 $$y_{ijk} = \mu + S_{i} + M_{j} + \left( {SM} \right)_{ij} + B\left( S \right)_{k\left( i \right)} + e_{ijk}$$

in which *y*_*ijk*_ is the observed arthropod damage, µ = the overall mean, *S*_*i*_ = the random site effect, *M*_*j*_ = the fixed material effect, (*SM*)_*ij*_ = the random interaction of material and site, *B*(*S*)_*k*(*i*)_ = the random replicate effect of within site, and *e*_*ijk*_ = the residual effect. Again SAS PROC MIXED was used separately for each arthropod damage endpoint in the analysis. The minimum and maximum mean values (reference range) were established from commercially available conventional reference hybrids to provide arthropod abundance or damage values representative of the natural variability within conventional maize for each arthropod.

#### Data interpretation method

Statistically significant differences between MON 87411 and conventional control were assessed for biological significance in the context of the range of the commercial reference hybrids, and for consistency with other collection methods, collection times (*S. frugiperda* only), and/or sites. Statistically significant differences for which MON 87411 mean values were within the reference range, or that were not consistently detected using multiple collection methods, or not consistently observed in environments in which the same arthropod taxa occurred, were not considered biologically meaningful in terms of adverse environmental impact.

## Results and discussion

An ERA of GM crops is conducted on a case-by-case basis using a weight of evidence approach and considering all relevant information. For the insect-protected GM crops, a step-wise, tiered testing approach using surrogate species is used since it is the recommended procedure to assess for potential effects on non-target organisms in many regulatory frameworks (Romeis et al. 2008, 2013). In the earliest tier, a battery of key arthropods with both agricultural and worldwide relevance is tested at doses of a test material (e.g. purified protein or dsRNA) well above those typically expressed in the plant. If the results of the first-tier studies require refinement, then higher-tiered testing may be conducted to address uncertainty in the risk assessment under progressively more realistic situations, and ultimately under field conditions if needed. In the case of insecticidal traits (DvSnf7 and Cry3Bb1) expressed in MON 87411, the tiered testing has not progressed beyond the early tiers due to the restricted activity spectrum of these traits (Palmer and Krueger 1999; Sinderman et al. 2002; Duan et al. 2008a, b; Li et al. 2008, 2010; Bachman et al. 2013). However, field studies to evaluate the effects of Cry3Bb1 on NTAs have been conducted and revealed no adverse effects to non-target arthropods (Ahmad et al. 2006; Bhatti et al. 2005a, b; Rauschen et al. 2010a, b; Rosca and Cagan 2012a, b; Svobodova et al. 2012a, b). To complement the portion of the NTA risk assessment focusing on adverse environmental effects, NTA field evaluations conducted as a part of plant characterization were also used to confirm findings from the lower-tier laboratory testing. We conducted a comprehensive field evaluation across three distinct geographic regions to understand how the NTAs that are most closely associated with the plant may respond to the introduction of MON 87411.

### NTA abundance

Across all regions, a total of 128 individual-site statistical comparisons were made between MON 87411 and the conventional control for arthropod abundance representing 20 taxa including: ant-like flower beetle, *Notoxus monodon* (Coleoptera: Anthicidae); aphid, several spp. (Homoptera: Aphididae); big-eyed bug, *Geocoris* spp. (Hemiptera: Geocoridae); corn flea beetle, *Chaetocnema pulicaria* (Coleoptera: Chrysomelidae); cornsilk fly, *Euxesta stigmatias* (Diptera: Otitidae); predatory earwig (Dermaptera: Forficulidae); delphacid planthopper (Delphacidae); lacewing, *Chrysoperla* spp. (Neuroptera: Chrysopidae); ladybird beetle, several spp. (Coleoptera: Coccinellidae); leafhopper, *Dalbulus maidis* (Homoptera: Cicadellidae); long-legged fly, *Dolichopus spp.* (Diptera: Dolichopodidae); *Maecolapsis* sp. (Coleoptera; Chrysomelidae); minute pirate bug, *Orius insidiosus* (Hemiptera: Anthocoridae); parasitic wasp, several spp. (Hymenoptera); predatory ground beetle, several spp. (Coleptera: Carabidae); sap beetle, several spp. (Coleoptera: Nitidulidae); shining flower beetle, *Phalacrus politus* (Coleoptera: Phalacridae); spider, several spp. (Araneae); spotted maize beetle, *Astylus atromaculatus* (Coleoptera: Melyridae); and hover fly, *Toxomerus* spp. (Syrphidae) (Tables 2, 3, 4). Lack of sufficient arthropod abundance precluded statistical comparisons between MON 87411 and the conventional control for 108 additional comparisons; however, descriptive statistics were provided for these comparisons (Supplementary material).

**Table 2:** Abundance of arthropods (Mean/Plot) determined using sticky traps and visual counts for MON 87411, conventional control, and references in 2012 US Field Trials

Arthropod^1^	Primary role	Site	Mean ± SE^2^		Reference range
			MON 87411	Control	
Sticky traps					
Aphid (Aphididae)	Herbivore	IABG	3.2 ± 0.95	1.2 ± 0.34	2.1–5.1
		NEYO	5.1 ± 1.03*	4.1 ± 1.41	4.9–7.5
Corn flea beetle (Chrysomelidae)	Herbivore	NCBD	1.1 ± 0.30	2.7 ± 0.88	1.2–14.4
		PAHM	5.7 ± 2.48	4.7 ± 1.21	6.5–15.6
Delphacid planthopper (Delphacidae)	Herbivore	IABG	0.7 ± 0.41	0.8 ± 0.35	1.4–1.8
		NCBD	15.4 ± 1.65	18.2 ± 1.95	23.2–30.3
		PAHM	4.6 ± 1.10	3.1 ± 0.82	2.4–8.3
Lacewing (Chrysopidae)	Predator	IABG	4.1 ± 0.53	4.4 ± 0.45	3.8–6.2
		NEYO	1.7 ± 0.30	1.1 ± 0.19	1.1–3.2
Ladybird beetle (Coccinellidae)	Predator	IABG	1.1 ± 0.17	1.0 ± 0.50	1.3 - 3.8
		NCBD	5.4 ± 0.78	6.5 ± 0.60	5.1–6.4
		PAHM	17.6 ± 3.00*	14.2 ± 1.10	13.1–16.8
Leafhopper (Cicadellidae)	Herbivore	NCBD	32.8 ± 4.42	35.9 ± 7.82	34.8–54.9
		PAHM	3.7 ± 0.87	3.9 ± 0.33	3.4–8.6
Minute pirate bug (Anthocoridae)	Predator	IABG	0.9 ± 0.42	1.6 ± 0.26	1.9–4.7
		NCBD	1.0 ± 0.24	0.7 ± 0.15	1.2–1.5
		PAHM	2.0 ± 0.53	2.2 ± 0.64	3.6–6.2
Parasitic wasp (Hymenoptera)	Parasitoid	IABG	32.9 ± 4.59	36.1 ± 3.82	38.6–76.4
		NCBD	89.4 ± 9.09	84.9 ± 12.02	122.7–139.0
		NEYO	12.7 ± 0.73	12.4 ± 0.93	12.6–30.4
		PAHM	122.3 ± 18.70	104.8 ± 5.66	116.0–159.5
Spider (Araneae)	Predator	NCBD	1.8 ± 0.14	1.5 ± 0.53	2.1–2.4
		PAHM	1.9 ± 0.52	0.9 ± 0.13	1.1–1.5
Visual counts					
Ant-like flower beetle (Anthicidae)	Pollen feeder	IABG	1.1 ± 0.21	2.4 ± 0.92	1.4–2.1
Corn flea beetle (Chrysomelidae)	Herbivore	NCBD	1.0 ± 0.44	1.8 ± 1.01	0.4–2.3
		PAHM	6.5 ± 1.38	5.8 ± 0.64	6.6–7.9
Ladybird beetle (Coccinellidae)	Predator	NCBD	1.7 ± 0.30	1.2 ± 0.05	1.1–1.7
		PAHM	2.3 ± 0.29	2.9 ± 0.93	1.5–1.8
Minute pirate bug (Anthocoridae)	Predator	IABG	2.1 ± 0.16	1.8 ± 0.26	1.4–2.3
		NCBD	1.2 ± 0.17	1.0 ± 0.8	0.8–1.4
		PAHM	4.4 ± 0.92	7.5 ± 1.25	4.7–7.8
Sap beetle (Nitidulidae)	Herbivore	IABG	6.6 ± 2.23	7.1 ± 3.14	2.3–5.0
		NCBD	3.3 ± 0.70	3.2 ± 0.85	3.1–4.5
		NEYO	2.7 ± 0.36	3.0 ± 0.43	1.3–1.6
		PAHM	4.1 ± 0.41	3.7 ± 1.16	2.4–4.2
Shining flower beetle (Phalacridae)	Pollen feeder	NCBD	4.4 ± 0.34	5.0 ± 1.02	3.4–6.2
		PAHM	1.3 ± 0.33	2.3 ± 0.13	1.1–1.7
Spider (Araneae)	Predator	NCBD	4.9 ± 1.13	3.9 ± 0.22	3.5–4.9
		PAHM	1.5 ± 0.58	1.2 ± 0.35	1.4–2.4

* Indicates statistically significant difference between MON 87411 and the conventional control (α = 0.05)

^1^Arthropods that met the minimum abundance criteria are included in the analysis

^2^ MON 87411 and the conventional control values represent means with standard error. N = 4

^3^ Reference range is calculated from the minimum and maximum mean values from among reference materials at each site

**Table 3:** Abundance of arthropods (Mean/Plot) determined using sticky traps and visual counts for MON 87411, conventional control, and references in 2012–2013 Argentina Field Trials

Arthropod^1^	Primary role	Site	Mean ± S.E.^2^		Reference range^3^
			MON 87411	Control	
Sticky traps					
Aphid (Aphididae)	Herbivore	BAGH	5.0 ± 0.61	5.5 ± 1.06	5.2–6.1
Lacewing (Chrysopidae)	Predator	BAFE	2.7 ± 0.56	1.9 ± 0.33	2.2–3.5
		BAGH	1.8 ± 0.57	1.5 ± 0.73	2.6–3.8
		ERMY	0.8 ± 0.24	1.0 ± 0.25	0.8–1.8
Ladybird beetle (Coccinellidae)	Predator	BAFE	5.9 ± 0.68	6.2 ± 0.99	3.8–7.9
		BAGH	1.8 ± 0 .25	1.7 ± 0.32	1.4–2.3
		TMBU	1.0 ± 0.08	0.9 ± 0.47	1.2–1.8
Leafhopper (Cicadellidae)	Herbivore	BAGH	6.5 ± 0.41	6.2 ± 0.50	7.7–11.8
		ERMY	2.6 ± 0.29	2.2 ± 0.60	2.0–3.9
		TMBU	23.7 ± 5.14	22.8 ± 6.37	19.0–25.6
Spotted maize beetle (Melyridae)	Herbivore	BAGH	1.1 ± 0.75	0.9 ± 0.36	0.3–3.2
		ERMY	1.4 ± 0.16	3.4 ± 2.37	1.1–2.9
		TMBU	8.1 ± 1.74	9.1 ± 1.16	8.7–11.2
Minute pirate bug (Anthocoridae)	Predators	BAFE	8.1 ± 0.54	6.4 ± 0.57	9.6–12.6
		BAGH	4.6 ± 0.74	5.5 ± 0.35	7.3–9.9
Parasitic wasp (Hymenoptera)	Parasitoid	ERMY	3.4 ± 0.50	2.9 ± 0.29	3.9–4.6
		TMBU	21.2 ± 2.72	17.1 ± 1.82	14.2–23.0
Sap beetle (Nitidulidae)	Herbivore	BAGH	2.1 ± 0.29	2.9 ± 0.58	2.5–3.8
		ERMY	2.4 ± 0.81	2.1 ± 0.24	1.3–3.7
		TMBU	1.3 ± 0.33	1.3 ± 0.09	1.3–2.0
Hover fly (Syrphidae)	Predator	BAGH	1.1 ± 0.09	1.1 ± 0.15	1.3–2.3
Visual counts					
Aphid (Aphididae)	Herbivore	BAFE	15.7 ± 8.90	6.5 ± 2.88	2.2–41.0
Predatory earwig (Forficulidae)	Predator	BAFE	35.2 ± 4.40	41.2 ± 3.95	23.7–41.8
		BAGH	16.0 ± 1.25	11.3 ± 1.22	12.5–17.7
		ERMY	2.0 ± 0.28	1.5 ± 0.25	1.2–1.9
		TMBU	22.8 ± 4.04	31.7 ± 2.01	21.8–35.8
Lacewing (Chrysopidae)	Predator	BAFE	2.6 ± 0.25	2.4 ± 0.31	2.0–3.3
		BAGH	1.3 ± 0.17	1.3 ± 0.16	1.0–1.4
		ERMY	1.4 ± 0.35	1.5 ± 0.12	1.5–2.8
		TMBU	1.0 ± 0.18	1.5 ± 0.32	1.1–1.8
Ladybird beetle (Coccinellidae)	Predator	BAFE	1.4 ± 0.79	1.0 ± 0.37	1.3–3.7
		TMBU	0.4 ± 0.08	1.0 ± 0.20	1.2–1.5
Spotted maize beetle (Melyridae)	Herbivore	BAFE	8.1 ± 3.40	2.8 ± 0.52	3.2–36.9
		BAGH	8.8 ± 3.43*	4.9 ± 1.42	3.8–7.6
		ERMY	2.3 ± 0.74	2.7 ± 0.82	0.5–14.7
		TMBU	7.6 ± 3.16	4.4 ± 0.82	4.1–12.6
Minute pirate bugs (Anthocoridae)	Predator	BAFE	10.4 ± 1.63	11.4 ± 1.07	9.3–13.3
		BAGH	7.8 ± 0.55	7.4 ± 0.55	7.8–9.4
		ERMY	3.1 ± 0.53	3.4 ± 0.63	2.3–3.6
		TMBU	3.2 ± 0.62	4.3 ± 0.81	3.8–5.0
Parasitic wasp (Hymenoptera)	Parasitoid	BAGH	3.3 ± 0.18	3.3 ± 0.48	3.2–3.8
Sap beetle (Nitidulidae)	Herbivore	BAFE	8.8 ± 0.73	8.6 ± 2.19	6.2–8.6
		BAGH	11.5 ± 1.79	11.3 ± 0.67	10.0–13.4
		ERMY	3.0 ± 0.69	3.8 ± 0.32	2.4–5.0
		TMBU	3.5 ± 1.43	2.3 ± 0.58	1.0–8.2
Spider (Araneae)	Predator	BAFE	3.0 ± 0.28	3.4 ± 0.57	2.7–3.4
		BAGH	2.1 ± 0.25	2.1 ± 0.20	1.7–2.5
		TMBU	3.8 ± 0.30	3.5 ± 0.29	3.8–5.8

* Indicates statistically significant difference between MON 87411 and the conventional control (α = 0.05)

^1^Arthropods that met the minimum abundance criteria are included in the analysis

^2^ MON 87411 and the conventional control values represent means with standard error. N = 4

^3^ Reference range is calculated from the minimum and maximum mean values from among reference materials at each site

**Table 4:** Abundance of Arthropods (Mean/Plot) Determined Using Sticky Traps and Visual Counts for MON 87411, Conventional Control, and References in 2013-14 Brazil Field Trials

Arthropod^1^	Primary role	Site	Mean ± S.E.^2^		Reference range^3^
			MON 87411	Control	
Sticky traps					
Big-eyed bug (Geocoridae)	Predator	SPSD	5.8 ± 1.21	4.0 ± 0.92	2.0–4.7
Cornsilk fly (Otitidae)	Herbivore	BALM	210.3 ± 33.77	244.5 ± 30.91	201.8–294.8
		MGCH	54.3 ± 2.56*	36.5 ± 2.63	38.3–47.3
		MTSO	377.1 ± 28.46	296.6 ± 18.40	232.7–327.8
		PRRO	244.7 ± 19.45	252.1 ± 21.70	139.9–336.3
		RSNM	291.7 ± 44.89	301.9 ± 29.78	172.3–219.6
		SPSD	230.8 ± 21.81	196.8 ± 15.32	163.0–227.8
Predatory earwig (Forficulidae)	Predator	MTSO	2.8 ± 0.63*	5.4 ± 2.14	2.5–5.0
		PRRO	6.6 ± 1.38	5.5 ± 1.49	4.8–6.6
		RSNM	3.3 ± 0.81	4.1 ± 0.44	3.3–5.2
Lacewing (Chrysopidae)	Predator	MGCH	1.0 ± 0.16	1.3 ± 0.09	0.9–1.5
Ladybird beetle (Coccinellidae)	Predator	MGCH	2.6 ± 0.36	2.4 ± 0.67	0.9–2.1
		SPSD	2.9 ± 0.88	2.6 ± 0.52	1.3–1.9
Leafhopper (Cicadellidae)	Herbivore	BALM	1008.6 ± 129.72	942.4 ± 93.50	851.5–1170
		MGCH	114.2 ± 2.53	108.6 ± 2.84	108.3–125.9
		MTSO	27.2 ± 3.45	21.1 ± 2.37	14.3–26.0
		PRRO	26.1 ± 1.08	31.3 ± 1.99	20.1–25.3
		RSNM	34.0 ± 4.44	40.7 ± 8.11	38.8–48.4
		SPSD	200.7 ± 22.24	164.2 ± 12.74	131.7–163.3
Long legged fly (Dolichopodidae)	Predator	PRRO	5.1 ± 1.45	5.8 ± 1.41	2.9–6.6
* Maecolapsis* sp. (Chrysomelidae)	Herbivore	MGCH	3.3 ± 0.40	3.3 ± 0.33	1.8–4.3
Minute pirate bug (Anthocoridae)	Predator	PRRO	4.7 ± 0.78	7.0 ± 0.95	3.4–6.6
		RSNM	17.2 ± 2.08	13.8 ± 2.66	9.8–15.1
		SPSD	9.5 ± 1.05	8.6 ± 1.81	5.7–7.1
Predatory ground beetle (Carabidae)	Predators	RSNM	2.7 ± 0.85	2.6 ± 0.43	0.7–2.0
Spider (Araneae)	Predator	MTSO	1.7 ± 0.31	2.5 ± 0.31	1.7–2.3
Visual counts					
Big-eyed bug (Geocoridae)	Predator	SPSD	1.9 ± 0.26	0.9 ± 0.19	0.9–1.4
Predatory earwig (Forficulidae)	Predator	BALM	0.9 ± 0.13	1.7 ± 0.64	1.2–2.4
		MGCH	3.0 ± 0.48	2.7 ± 0.34	2.9–3.6
		MTSO	2.4 ± 0.54	2.6 ± 0.09	3.5–4.3
		PRRO	13.5 ± 1.32	13.3 ± 1.28	14.0–14.8
		RSNM	4.0 ± 0.22	4.4 ± 0.54	4.0–5.3
		SPSD	2.4 ± 0.75	1.9 ± 0.21	1.9–3.2
Ladybird beetle (Coccinellidae)	Predator	SPSD	1.4 ± 0.09	0.9 ± 0.15	1.2–1.4
Minute pirate bug (Anthocoridae)	Predator	RSNM	1.6 ± 0.32	1.8 ± 0.49	1.9–2.4
		SPSD	2.7 ± 0.35	1.2 ± 0.34	1.0–2.2
Sap beetle (Nitidulidae)	Herbivore	MTSO	6.8 ± 0.27	6.0 ± 0.27	6.0–7.6
		PRRO	9.3 ± 0.64	6.3 ± 0.50	6.3–18.6
		SPSD	7.3 ± 0.64	6.3 ± 0.50	3.9–6.5
Spider (Araneae)	Predator	MGCH	1.3 ± 0.38	1.1 ± 0.31	1.0–1.2
		SPSD	3.0 ± 0.40	2.1 ± 0.05	2.5–2.7

* Indicates statistically significant difference between MON 87411 and the conventional control (α = 0.05)

^1^Arthropods that met the minimum abundance criteria are included in the analysis

^2^ MON 87411 and the conventional control values represent means with standard error. N = 4

^3^ Reference range is calculated from the minimum and maximum mean values from among reference materials at each site

Across all three regions, no statistically significant differences were detected between MON 87411 and the conventional control for 123 of the 128 comparisons (96.1 %). In the U.S., statistically significant differences were detected in two taxa; aphid and ladybird beetle (Table 2). The mean abundance of aphids associated with MON 87411 was higher than the conventional control at one of the two sites where aphids were observed (*P* = 0.0132). However, the mean value for aphid abundance associated with MON 87411 was within the range of the commercial reference hybrids (MON 87411 mean = 5.1 per plot; reference range 4.9–7.5 per plot). The mean abundance of ladybird beetles was higher for MON 87411 than the conventional control at one of the three sites where ladybird beetles were observed. The mean abundance value for ladybird beetles associated with MON 87411 was slightly outside the range of the commercial reference hybrids (MON 87411 mean = 17.6 per plot; reference range 13.1–16.8 per plot). In Argentina, a single statistically significant difference was detected where MON 87411 had higher spotted maize beetle abundance compared to the conventional control at one of the four sites where spotted maize beetle was observed (*P* = 0.0345) (Table 3). The mean abundance value for spotted maize beetle on MON 87411 was slightly outside the range of the commercial reference hybrids (MON 87411 mean = 8.8 per plot; reference range 3.8–7.6 per plot). In Brazil, statistically significant differences were detected in two taxa; cornsilk fly and predatory earwig (Table 4). The mean abundance of cornsilk fly associated with MON 87411 was higher than the conventional control at one of the six sites where cornsilk fly was observed (*P* = 0.0014). The mean abundance value for cornsilk fly associated with MON 87411 was slightly outside the range of the commercial reference hybrids (MON 87411 mean = 54.3 per plot; reference range 38.3–47.3 per plot). The mean abundance of predatory earwig was lower for MON 87411 than the conventional control at one of the three sites where predatory earwig was observed. (P = 0.005). However, the mean value of predatory earwig abundance on MON 87411 was within the range of commercial reference hybrids (MON 87411 mean = 2.8 per plot; reference range 2.5–5.0 per plot).

In each case where no differences were detected or where differences were detected in NTA abundance, the mean value for MON 87411 was within the reference range and/or the difference was not consistently observed across collection methods and/or sites. Thus, these differences were not indicative of a consistent response associated with the trait and are not considered biologically meaningful in terms of adverse environmental impact of MON 87411 compared to conventional maize.

A high degree of similarity of taxa across regions was observed especially for the most abundant taxa representing the ecological functions of herbivores, predators and parasitoids in maize fields: aphid, predatory earwig, lacewing, ladybird beetle, leafhopper, minute pirate bug, parasitic wasp, sap beetle, and spider. For the nine widely distributed taxa, no statistically significant differences in their abundance were detected between MON 87411 and the conventional control (Table 5). A retrospective power analysis of the data indicated that population-level effects of 50 % were detectable with 80 % power for the widely distributed taxa across regions (Table 5). Therefore, given the scale and intensity of the sampling, any significant impacts of MON 87411 maize on populations of widely distributed taxa should have been detectable within this study.

**Table 5:** Abundance of Arthropods (Mean/Plot) Associated with MON 87411 and the Conventional Control in Field Trials Across Regions

Arthropod^1^	Number of Regions	Number of Sites across Regions	Mean		*P* Value	Statistical Power (%)^2^
			MON 87411	Control		
Aphid (Aphididae)	2	4	7.2	4.3	0.279	73.1
Predatory earwig (Forficulidae)	2	10	12.0	13.3	0.194	100.0
Lacewing (Chrysopidae)	3	6	2.3	2.3	0.956	87.3
Ladybird beetle (Coccinellidae)	3	8	4.9	4.6	0.496	100.0
Leafhopper (Cicadellidae)	3	11	103.7	96.5	0.615	100.0
Minute pirate bug (Anthocoridae)	3	10	8.1	8.2	0.990	100.0
Parasitic wasp (Hymenoptera)	2	7	38.8	35.8	0.242	100.0
Sap beetle (Nitidulidae)	3	11	6.6	6.7	0.778	100.0
Spider (Araneae)	3	8	3.1	2.8	0.355	99.5

^1^ Arthropods observed that were most abundant and similar across regions

^2^ Statistical power to detect a 50 % difference in abundance between MON 87411 and control

### NTA damage

A total of 56 statistical comparisons were made between MON 87411 and the conventional control for plant damage caused by the following non-target arthropods: *O. nubilalis*, *D. saccharalis*, *H. zea*, and *S. frugiperda*. Across all three regions, no statistically significant differences were detected between MON 87411 and the conventional control for 53 of the 56 comparisons (94.6 %) (Tables 6, 7, 8, 9). Lack of variability in the data precluded statistical comparisons between MON 87411 and conventional control for one additional comparison; however, the mean for MON 87411 and the conventional control were the same value for this comparison, indicating no biological differences.

**Table 6:** Non-Target Arthropod Pest Damage to MON 87411, Conventional Control, and References in 2012 U.S. Field Trials

Non-target arthropod pest	Damage assessment	Site	Mean ± S.E.^1^		Reference range^2^
			MON 87411	Control	
*H*. *zea*	Ear damage area of 10 plants per plot (cm^2^)	IABG	0.7 ± 0.38	0.5 ± 0.28	0.5–1.3
		NCBD	3.3 ± 1.25*	1.5 ± 0.39	0.7–1.8
		NEYO	3.2 ± 0.13	3.0 ± 0.22	2.3–3.2
		PAHM	0.3 ± 0.23	0.2 ± 0.11	0.2–0.3
*O*. *nubilalis*	Number of stalk galleries of 10 plants per plot	IABG	0.0 ± 0.03	0.0 ± 0.00	0.0–0.1
		NCBD	0.1 ± 0.03	0.1 ± 0.04	0.1–0.3
		NEYO	0.0 ± 0.00	0.0 ± 0.00	0.0–0.0
		PAHM	1.4 ± 0.24	1.8 ± 0.33	1.4–1.8
*O*. *nubilalis*	Stalk gallery length (cm) of 10 plants per plot	IABG	0.1 ± 0.08	0.0 ± 0.00	0.0–0.3
		NCBD	0.5 ± 0.19	0.2 ± 0.14	0.5–0.7
		NEYO	0.0 ± 0.00	0.0 ± 0.00	0.0–0.1
		PAHM	5.9 ± 1.46	7.9 ± 1.46	5.6–8.3

* Indicates a significant difference between MON 87411 and the conventional control (α = 0.05) using ANOVA

^1^ MON 87411 and the conventional control values represent means with standard error in parentheses

^2^ Reference range is calculated from the minimum and maximum mean values from among four reference materials at each site

**Table 7:** Non-target arthropod pest damage to MON 87411, conventional control, and references in 2012–2013 Argentina Field Trials

Non-Target Arthropod Pest	Damage assessment	Site	Mean ± S.E.^1^		Reference range^2^
			MON 87411	Control	
*H*. *zea*	Ear damage area of 10 plants per plot (cm^2^)	BAFE	6.0 ± 0.83	6.1 ± 0.53	4.8–5.8
		BAGH	14.2 ± 1.32	16.3 ± 0.40	8.0–18.7
		ERMY	1.0 ± 0.39	1.0 ± 0.28	0.4–1.1
		TMBU	1.0 ± 0.25	1.0 ± 0.32	0.7–1.3
*D. saccharalis*	Number of stalk galleries of 10 plants per plot	BAFE	3.5 ± 0.99	4.4 ± 0.53	2.9–4.2
		BAGH	2.6 ± 0.28	3.0 ± 0.44	2.3–3.0
		ERMY	1.5 ± 0.16	1.1 ± 0.30	1.1–1.5
		TMBU	0.3 ± 0.16	0.3 ± 0.09	0.2–0.5
*D. saccharalis*	Stalk gallery length (cm) of 10 plants per plot	BAFE	23.3 ± 6.52	27.9 ± 4.24	19.4–26.2
		BAGH	15.6 ± 2.86	17.6 ± 5.31	12.9–19.3
		ERMY	13.7 ± 3.21	9.8 ± 2.84	6.7–12.2
		TMBU	2.3 ± 1.36	2.8 ± 1.09	1.3–5.5

**Table 8:** Non-target arthropod pest damage to MON 87411, conventional control, and references in 2012–2013 Argentina Field Trials

Non-target arthropod pest	Damage assessment	Site	Observation number	Mean ± S.E.^1^		Reference range^2^
				MON 87411	Control	
*S. frugiperda*	Damage area of 10 plants per plot (rating 0-9)	BAFE	1	0.3 ± 0.21	0.2 ± 0.14	1.0–1.6
			2	0.5 ± 0.28	0.2 ± 0.11	0.4–1.0
			3	0.5 ± 0.17	0.6 ± 0.19	0.3–0.6
			4	0.2 ± 0.13	0.1 ± 0.05	0.1–0.3
			5	0.2 ± 0.10	0.1 ± 0.08	0.0–0.1
		BAGH	1	0.6 ± 0.33	0.5 ± 0.30	1.6–1.8
			2	0.0 ± 0.00^†^	0.0 ± 0.00^†^	0.0–0.0
		ERMY	1	1.5 ± 0.38	1.6 ± 0.72	1.9–3.0
			2	4.1 ± 0.39	3.4 ± 0.38	3.6–5.7
			3	4.9 ± 0.36	3.6 ± 0.50	2.5–4.1
			4	3.4 ± 0.80	3.5 ± 1.15	1.7–3.9
			5	3.9 ± 0.96	2.8 ± 0.64	1.7–2.7
		TMBU	1	0.2 ± 0.11	0.2 ± 0.08	0.4–1.4
			2	1.5 ± 0.22	1.6 ± 0.24	1.9–2.3
			3	1.5 ± 0.30*	2.2 ± 0.17	1.3–2.0

**Table 9:** Non-target arthropod pest damage to MON 87411, conventional control, and references in 2013–2014 Brazil Field Trials

Non-target arthropod pest	Damage assessment	Site	Mean ± S.E.^1^		Reference range^2^
			MON 87411	Control	
*H*. *zea* and *S. frugiperda*	Ear damage area of 10 plants per plot (cm^2^)	BALM	5.3 ± 0.96	4.6 ± 1.52	0.5–1.6
		MGCH	1.0 ± 0.29	0.8 ± 0.35	0.0–1.1
		MTSO	1.2 ± 0.36	1.1 ± 0.31	0.3–0.7
		PRRO	2.3 ± 0.72	3.4 ± 0.31	1.8–4.1
		RSNM	10.3 ± 1.31	11.5 ± 1.59	4.9–9.7
		SPSD	1.9 ± 0.36*	3.3 ± 0.43	1.0–1.7
*S. frugiperda*	Damage area of 10 plants per plot (rating 0-9)	BALM	7.1 ± 0.66	5.9 ± 0.54	4.8–6.8
		MGCH	0.8 ± 0.35	0.6 ± 0.17	0.4–0.7
		MTSO	2.9 ± 0.19	3.3 ± 0.42	2.6–3.2
		PRRO	3.0 ± 0.42	2.6 ± 0.19	2.2–2.5
		RSNM	0.8 ± 0.41	1.4 ± 0.46	0.7–2.4
		SPSD	3.6 ± 0.22	4.1 ± 0.41	4.0–4.5
*D. saccharalis*	Stalk gallery length (cm) of 10 plants per plot	BALM	1.0 ± 0.36	0.7 ± 0.23	0.3–1.0
		MGCH	1.3 ± 0.34	1.6 ± 0.69	3.3–12.9
		MTSO	37.7 ± 3.57	35.4 ± 5.09	25.1–45.7
		PRRO	2.0 ± 1.41	0.0 ± 0.00	0.7–4.2
		RSNM	0.3 ± 0.17	0.1 ± 0.050	0.0–0.8
		SPSD	0.3 ± 0.18	0.3 ± 0.28	0.0–0.3

* Indicates a significant difference between MON 87411 and the conventional control (α = 0.05) using ANOVA

^1^ MON 87411 and the conventional control values represent means with standard error in parentheses

^2^ Reference range is calculated from the minimum and maximum mean values from among four reference materials at each site

A total of three statistically significant differences involving two taxa were detected between MON 87411 and conventional control. In the U.S., MON 87411 had higher ear damage than conventional control from *H. zea* at one of the four sites (*P* < 0.05) (Table 6). In Argentina, MON 87411 had less leaf damage than conventional control from *S. frugiperda* in the third observation at one of the four sites (*P* < 0.05) (Tables 7, 8). In Brazil, MON 87411 had less ear damage than the conventional control from *H. zea* and *S. frugiperda* at one of the six sites (*P* < 0.05) (Table 9). In each case where a significant difference in NTA damage between MON 87411 and the conventional control was detected, mean values for MON 87411 were within the reference range and/or difference between test and control were not consistently observed across observation times and/or sites (Tables 6, 7, 8, 9). Thus, these differences were not indicative of consistent plant responses associated with the insect-protected and glyphosate tolerant traits and are unlikely to be biologically meaningful in terms of increased adverse environmental impact of MON 87411 compared to conventional maize.

### Representative taxa and data transportability

This study was conducted in diverse maize growing regions representative of temperate and tropical agro-ecological zones and assessed representative arthropods consistent with the representative taxa concept and surrogate species approach used for the NTA risk assessment of GM crops. The taxa evaluated were appropriate for use in this study because they had the potential for direct or indirect exposure to the trait, were sufficiently abundant, and were relevant for risk assessment (Garcia-Alonso et al. 2006; Rose 2006; Romeis et al. 2008, 2009, 2013). Since it is not practically possible to evaluate all the arthropods during field evaluation of NTAs, a “representative taxa concept” was utilized to focus on those taxa for which data can be reliably obtained and statistical robustness can be guaranteed (Knecht et al. 2010; Albajes et al. 2013; Carstens et al. 2014). The two most commonly used criteria for selection of representative taxa are consistency in abundance over the typical geographic range of the crop, and the suitability of taxa to detect small differences between the GM crop and its conventional comparator (Meissle et al. 2013; Albajes et al. 2013; Comas et al. 2013, 2014, 2015). In this study, we also provided further evidence for the adoption of the representative taxa concept for use in the environmental risk assessment of GM crops.

In the current assessments of NTA abundance, twenty arthropod taxa met minimum abundance criteria for statistical analysis. Nine of these taxa occurred in at least two of the three regions and in at least four sites across regions: aphid, predatory earwig, lacewing, ladybird beetle, leafhopper, minute pirate bug, parasitic wasp, sap beetle, and spider. In addition to wide regional distribution, these nine taxa fit the concept of representative taxa for field tests evaluating the impact of insect-protected maize on NTAs and encompass the ecological functions of herbivores, predators and parasitoids that would typically be subjected to above ground exposure of these traits. The nine taxa we identified also meet the recommendations of Knecht et al. (2010), Albajes et al. (2013), and Comas et al. (2013 and 2015) on abundance consistency and capacity to detect potential effects. A similar concept, the surrogate species approach has been used for tier 1 laboratory studies where indicator organisms are selected as representative taxa for hazard testing in an ERA (Garcia-Alonso et al. 2006; Romeis et al. 2011; Carstens et al. 2014). Surrogate species are typically chosen due to their relevance to the crop and amenability to testing in micro-environments (Barrett et al. 1994; Rose 2006; Romeis et al. 2008). The use of the surrogate species approach has allowed laboratory data generated on the effects of insecticidal traits on NTAs in one region, to be used in different regions, without necessarily repeating these studies.

Therefore, the nine taxa identified in our studies may serve as representative taxa in maize agro-ecosystems, indicating that the data are readily transportable for use in risk assessment between these geographic regions and to other regions with similar fauna. The beneficial impact of transportable data based on the similarity of NTAs in commercial maize-growing regions indicates that repeated local field trials may not be necessary and may represent duplicated effort with limited value for the ERA of a GM crop. The few differences in taxa that may occur across geographies are not barriers to data transportability but require appropriate consideration in the context of problem formulation and tiered testing in the ERA.

## Conclusion

Leveraging relevant transportable data across geographies for the ERA of GM crops can provide useful pertinent data to risk assessors and may result in significant time and cost savings by eliminating duplicated field work (Garcia-Alonso et al. 2014; Horak et al. 2015a, b; Nakai et al. 2015). Irrespective of variations in climate, region, and overall biodiversity of a given region, our results indicate high similarity across regions for important functional groups represented by herbivores, predatory and parasitic arthropod taxa closely associated with maize within agroecosystems where the crop is grown. This high degree of similarity of taxa across regions indicates that findings from one region are relevant, and thus transportable for use in the ERA of similar GM crop products in other regions.

The results of the NTA assessments in multi-site and multi-region field trials demonstrate the absence of adverse effects when NTA communities are exposed to maize MON 87411 expressing DvSnf7, Cry3Bb1, and CP4 EPSPS traits. Our results are in agreement with other studies that demonstrate the absence of adverse effects independently for Dvsnf7 (Bachman et al. 2013), Cry3Bb1 (Lundgren and Wiedenmann 2002; Al-Deeb and Wilde 2003; Ahmad et al. 2005, 2006; Bhatti et al. 2005a, b; Li and Romeis 2009, 2011; Devos et al. 2012; Comas et al. 2014; ILSI-CERA 2014), and CP4 EPSPS (Reyes 2005; Rosca 2004; Schier 2006; ILSI-CERA 2010; Comas et al. 2014). These field results confirm findings from the lower-tier laboratory testing by demonstrating no adverse effect on arthropod communities representing the ecological functions of herbivores, predators, and parasitoids in maize agro-ecosystems. Additionally, these NTA assessments provide further support for the extrapolation of laboratory results to the field.

Field data on NTAs obtained in this study for a CRW-protected GM maize were similar across diverse geographic regions in arthropod taxa representative of ecologically relevant taxonomic and functional groups. Therefore, along with pertinent laboratory data, appropriate plant characterization and NTA field data are relevant and transportable to other geographies for the ERA of the same GM crop, or related traits or GM crop/trait combinations where the ecological assessment endpoints are similar. It is important that regulators have access to and utilize environmental assessment data on the crop and trait that are generated in other geographies. Leveraging existing, relevant data for the ERA of GM crops across geographies will conserve resources, eliminate redundancy, and support conclusions with high certainty for assessing potential environmental risk from the commercial release of a GM crop.

## Electronic supplementary material

Supplementary material 1 (DOCX 26 kb)
