# Supplementary material for: Transportable data from non-target arthropod field studies for the environmental risk assessment of genetically modified maize expressing an insecticidal double-stranded RNA
Source: Transgenic Res. 2015 Oct 3;25:1–17. doi: 10.1007/s11248-015-9907-3 (PMC4735227; doi:10.1007/s11248-015-9907-3)
Supplement: Supplementary file 1 — Supplementary material 1 (DOCX 26 kb) [file 11248_2015_9907_MOESM1_ESM.docx]

**Appendix 1.** Abundance summary for sites and arthropods below the inclusion criterion and were excluded from significance testing. Total counts by collection method, arthropod taxa, site, and material over collection times and replicates. The last two columns are the total and mean count across all collection times per plot over all materials.

| **Collection Method** | **Arthropod** | **Region** | **Site^1^** | **Total Counts** | | | **Total** | **Mean / Plot** |
| --- | --- | --- | --- | --- | --- | --- | --- | --- |
|  |  |  |  | **MON 87411** | **Control** | **Reference Range (min-max)** |  |  |
| Sticky | Aphid | U.S. | NCBD | 21 | 10 | 4 - 46 | 133 | 0.95 |
| Sticky | Aphid | U.S. | PAHM | 12 | 9 | 3 - 15 | 73 | 0.61 |
| Sticky | Assassin bug | Brazil | MGCH | 0 | 0 | 0 - 2 | 3 | 0.03 |
| Sticky | Big-eyed bug | Brazil | BALM | 0 | 1 | 0 - 0 | 1 | 0.01 |
| Sticky | Big-eyed bug | Brazil | MGCH | 0 | 1 | 0 - 0 | 1 | 0.01 |
| Sticky | Big-eyed bug | Brazil | MTSO | 2 | 2 | 0 - 1 | 5 | 0.04 |
| Sticky | Big-eyed bug | Brazil | PRRO | 0 | 8 | 3 - 9 | 30 | 0.25 |
| Sticky | Corn flea beetle | U.S. | IABG | 7 | 7 | 5 - 31 | 95 | 0.68 |
| Sticky | Corn flea beetle | U.S. | NEYO | 2 | 0 | 0 - 3 | 8 | 0.06 |
| Sticky | Damsel bug | Brazil | BALM | 17 | 6 | 4 - 15 | 59 | 0.49 |
| Sticky | Damsel bug | Brazil | SPSD | 7 | 3 | 1 - 7 | 26 | 0.22 |
| Sticky | Delphacid planthopper | U.S. | NEYO | 4 | 3 | 2 - 3 | 20 | 0.36 |
| Sticky | Predatory earwig | Argentina | BAGH | 2 | 0 | 1 - 4 | 14 | 0.58 |
| Sticky | Predatory earwig | Brazil | BALM | 8 | 20 | 8 - 33 | 110 | 0.92 |
| Sticky | Predatory earwig | Brazil | MGCH | 23 | 15 | 9 - 19 | 94 | 0.78 |
| Sticky | Predatory earwig | Brazil | SPSD | 2 | 1 | 2 - 4 | 16 | 0.13 |
| Sticky | Grasshopper | U.S. | PAHM | 0 | 0 | 0 - 1 | 1 | 0.04 |
| Sticky | Lacewing | Argentina | TMBU | 1 | 2 | 3 - 6 | 22 | 0.18 |
| Sticky | Lacewing | Brazil | BALM | 18 | 11 | 8 - 22 | 87 | 0.73 |
| Sticky | Lacewing | Brazil | MTSO | 5 | 6 | 5 - 13 | 48 | 0.40 |
| Sticky | Lacewing | Brazil | PRRO | 2 | 0 | 0 - 1 | 3 | 0.03 |
| Sticky | Lacewing | Brazil | RSNM | 1 | 4 | 1 - 6 | 18 | 0.15 |
| Sticky | Lacewing | Brazil | SPSD | 4 | 12 | 2 - 12 | 39 | 0.33 |
| Sticky | Lacewing | U.S. | NCBD | 2 | 4 | 0 - 2 | 13 | 0.23 |
| Sticky | Lacewing | U.S. | PAHM | 2 | 1 | 0 - 3 | 7 | 0.15 |
| Sticky | Ladybird beetle | Argentina | ERMY | 17 | 23 | 11 - 25 | 113 | 0.95 |
| Sticky | Ladybird beetle | Brazil | BALM | 16 | 13 | 6 - 14 | 67 | 0.56 |
| Sticky | Ladybird beetle | Brazil | MTSO | 11 | 2 | 10 - 18 | 72 | 0.60 |
| Sticky | Ladybird beetle | Brazil | PRRO | 1 | 4 | 1 - 12 | 22 | 0.18 |
| Sticky | Ladybird beetle | Brazil | RSNM | 2 | 0 | 0 - 4 | 8 | 0.07 |
| Sticky | Ladybird beetle | U.S. | NEYO | 6 | 10 | 7 - 38 | 98 | 0.70 |
| Sticky | Leaf-footed bug | Brazil | SPSD | 1 | 1 | 1 - 5 | 11 | 0.09 |
| Sticky | Leafhopper | U.S. | IABG | 1 | 1 | 1 - 2 | 8 | 0.06 |
| Sticky | Minute pirate bug | Argentina | ERMY | 2 | 1 | 0 - 1 | 5 | 0.04 |
| Sticky | Minute pirate bug | Argentina | TMBU | 19 | 18 | 16 - 25 | 112 | 0.93 |
| Sticky | Minute pirate bug | Brazil | BALM | 0 | 0 | 0 - 1 | 1 | 0.01 |
| Sticky | Minute pirate bug | Brazil | MTSO | 0 | 1 | 0 - 1 | 2 | 0.02 |
| Sticky | Minute pirate bug | U.S. | NEYO | 5 | 0 | 3 - 6 | 23 | 0.16 |
| Sticky | Sap beetle | U.S. | IABG | 0 | 1 | 0 - 5 | 10 | 0.18 |
| Sticky | Sap beetle | U.S. | NCBD | 14 | 9 | 4 - 13 | 69 | 0.82 |
| Sticky | Sap beetle | U.S. | PAHM | 3 | 4 | 2 - 10 | 34 | 0.71 |
| Sticky | Spider | Argentina | BAGH | 11 | 19 | 14 - 18 | 93 | 0.78 |
| Sticky | Spider | Argentina | ERMY | 4 | 2 | 9 - 13 | 50 | 0.42 |
| Sticky | Spider | Argentina | TMBU | 3 | 5 | 1 - 3 | 17 | 0.14 |
| Sticky | Spider | Brazil | BALM | 0 | 0 | 0 - 1 | 1 | 0.01 |
| Sticky | Spider | Brazil | MGCH | 17 | 14 | 14 - 17 | 92 | 0.77 |
| Sticky | Spider | Brazil | PRRO | 17 | 17 | 17 - 26 | 117 | 0.98 |
| Sticky | Spider | Brazil | RSNM | 0 | 1 | 1 - 5 | 11 | 0.09 |
| Sticky | Spider | Brazil | SPSD | 14 | 5 | 12 - 21 | 83 | 0.69 |
| Sticky | Spider | U.S. | IABG | 7 | 4 | 3 - 13 | 49 | 0.35 |
| Sticky | Spider | U.S. | NEYO | 4 | 2 | 2 - 7 | 29 | 0.21 |
| Sticky | Rove beetle | Argentina | BAFE | 2 | 4 | 1 - 4 | 18 | 0.75 |
| Sticky | Syrphidae | Brazil | MGCH | 3 | 3 | 0 - 5 | 20 | 0.17 |
| Sticky | Syrphidae | Brazil | PRRO | 20 | 22 | 14 - 23 | 116 | 0.97 |
| Sticky | Syrphidae | Brazil | SPSD | 7 | 13 | 9 - 17 | 67 | 0.56 |
| Visual | Big-eyed bug | Brazil | BALM | 0 | 0 | 0 - 1 | 1 | 0.01 |
| Visual | Big-eyed bug | Brazil | PRRO | 5 | 2 | 3 - 9 | 32 | 0.27 |
| Visual | Click beetle | U.S. | NCBD | 11 | 15 | 10 - 17 | 95 | 0.68 |
| Visual | Damsel bug | Argentina | BAFE | 0 | 2 | 0 - 1 | 3 | 0.13 |
| Visual | Grasshopper | Argentina | ERMY | 0 | 0 | 0 - 1 | 1 | 0.04 |
| Visual | Japanese beetle | U.S. | NCBD | 1 | 0 | 0 - 1 | 3 | 0.02 |
| Visual | Lacewing | Brazil | BALM | 3 | 0 | 1 - 3 | 11 | 0.09 |
| Visual | Lacewing | Brazil | MGCH | 0 | 1 | 0 - 4 | 10 | 0.08 |
| Visual | Lacewing | Brazil | MTSO | 1 | 0 | 0 - 2 | 5 | 0.04 |
| Visual | Lacewing | Brazil | RSNM | 1 | 1 | 1 - 2 | 8 | 0.07 |
| Visual | Lacewing | Brazil | SPSD | 18 | 18 | 11 - 17 | 94 | 0.78 |
| Visual | Lacewing | U.S. | IABG | 2 | 0 | 2 - 7 | 23 | 0.21 |
| Visual | Lacewing | U.S. | NCBD | 8 | 11 | 4 - 5 | 41 | 0.29 |
| Visual | Lacewing | U.S. | NEYO | 3 | 2 | 1 - 4 | 17 | 0.12 |
| Visual | Lacewing | U.S. | PAHM | 4 | 2 | 5 - 10 | 43 | 0.36 |
| Visual | Ladybird beetle | Argentina | BAGH | 10 | 10 | 8 - 12 | 62 | 0.43 |
| Visual | Ladybird beetle | Argentina | ERMY | 0 | 1 | 0 - 3 | 8 | 0.06 |
| Visual | Ladybird beetle | Brazil | BALM | 1 | 0 | 0 - 3 | 8 | 0.07 |
| Visual | Ladybird beetle | Brazil | MGCH | 5 | 2 | 6 - 12 | 44 | 0.37 |
| Visual | Ladybird beetle | Brazil | MTSO | 7 | 3 | 3 - 8 | 31 | 0.26 |
| Visual | Ladybird beetle | Brazil | PRRO | 0 | 2 | 1 - 6 | 11 | 0.09 |
| Visual | Ladybird beetle | Brazil | RSNM | 0 | 0 | 0 - 1 | 1 | 0.01 |
| Visual | Ladybird beetle | U.S. | IABG | 2 | 1 | 1 - 4 | 15 | 0.13 |
| Visual | Ladybird beetle | U.S. | NEYO | 0 | 0 | 0 - 1 | 2 | 0.01 |
| Visual | Lygus | U.S. | PAHM | 4 | 9 | 1 - 6 | 32 | 0.33 |
| Visual | Maize weevil | Brazil | MGCH | 3 | 2 | 0 - 0 | 5 | 0.04 |
| Visual | Minute pirate bug | Brazil | BALM | 1 | 1 | 0 - 2 | 6 | 0.05 |
| Visual | Minute pirate bug | Brazil | MGCH | 3 | 0 | 0 - 2 | 6 | 0.05 |
| Visual | Minute pirate bug | Brazil | MTSO | 0 | 0 | 0 - 1 | 3 | 0.03 |
| Visual | Minute pirate bug | Brazil | PRRO | 10 | 10 | 4 - 14 | 62 | 0.52 |
| Visual | Predatory ground beetle | Argentina | TMBU | 7 | 19 | 13 - 22 | 94 | 0.65 |
| Visual | Predatory ground beetle | Brazil | PRRO | 3 | 3 | 1 - 4 | 18 | 0.15 |
| Visual | Sap beetle | Brazil | RSNM | 15 | 5 | 7 - 23 | 72 | 0.60 |
| Visual | Spider | Argentina | ERMY | 12 | 12 | 4 - 6 | 44 | 0.31 |
| Visual | Spider | Brazil | BALM | 0 | 0 | 0 - 2 | 2 | 0.02 |
| Visual | Spider | Brazil | MTSO | 3 | 2 | 1 - 8 | 24 | 0.20 |
| Visual | Spider | Brazil | PRRO | 16 | 30 | 8 - 24 | 102 | 0.85 |
| Visual | Spider | Brazil | RSNM | 9 | 10 | 2 - 22 | 60 | 0.50 |
| Visual | Spider | U.S. | IABG | 10 | 12 | 3 - 16 | 68 | 0.61 |
| Visual | Spider | U.S. | NEYO | 2 | 3 | 1 - 7 | 25 | 0.18 |
| Visual | Rove beetle | Argentina | BAFE | 0 | 0 | 0 - 1 | 1 | 0.04 |
| Visual | Rove beetle | Brazil | MGCH | 7 | 6 | 3 - 11 | 39 | 0.33 |
| Visual | Stink bug | U.S. | IABG | 4 | 5 | 2 - 6 | 32 | 0.29 |
| Visual | Stink bug | U.S. | NCBD | 11 | 8 | 2 - 14 | 71 | 0.51 |
| Visual | Stink bug | U.S. | NEYO | 0 | 2 | 0 - 3 | 8 | 0.06 |
| Visual | Stink bug | U.S. | PAHM | 0 | 1 | 0 - 24 | 29 | 0.24 |

^1^ Site code: IABG = Greene County, IA; NCBD = Perquimans County, NC; NEYO = York County, NE; PAHM = Berks County, PA. BAFE = Ferré, Buenos Aires; BAGH = Gahan, Buenos Aires; ERMY = Montoya, Entre Ríos; TMBU = Burruyacú, Tucumán; BALM = Luis Eduardo Magalhães, BA; MGCH = Cachoeira Dourada, MG; MTSO = Sorriso, MT; PRRO = Rolândia, PR; RSNM = Não-Me-Toque, RS; SPSD = Santa Cruz das Palmeiras, SP.
